# A Phospholipid-Protein Complex from Antarctic Krill Reduced Plasma Homocysteine Levels and Increased Plasma Trimethylamine-*N*-Oxide (TMAO) and Carnitine Levels in Male Wistar Rats

**DOI:** 10.3390/md13095706

**Published:** 2015-09-08

**Authors:** Bodil Bjørndal, Marie S. Ramsvik, Carine Lindquist, Jan E. Nordrehaug, Inge Bruheim, Asbjørn Svardal, Ottar Nygård, Rolf K. Berge

**Affiliations:** 1 Department of Clinical Science, University of Bergen, 5020 Bergen, Norway; E-Mails: Bodil.Bjorndal@uib.no (B.B.); Marie.Ramsvik@olympic.no (M.S.R.); Carine.Lindquist@uib.no (C.L.); jan.erik.nordrehaug@sus.no (J.E.N.); Asbjorn.Svardal@uib.no (A.S.); Ottar.Nygard@helse-bergen.no (O.N.); 2 Olympic Seafood AS, N-6080 Fosnavaag, Norway; E-Mail: Inge.Bruheim@olympic.no; 3 Department of Cardiology, Stavanger University Hospital, 4036 Stavanger, Norway; 4 Department of Heart Disease, Haukeland University Hospital, 5021 Bergen, Norway

**Keywords:** phospholipid-protein complex, Omega-3 polyunsaturated fatty acids, *Euphausia superba*, one-carbon metabolism, homocysteine, trimethylamine-*N*-oxide, carnitine

## Abstract

Seafood is assumed to be beneficial for cardiovascular health, mainly based on plasma lipid lowering and anti-inflammatory effects of *n*-3 polyunsaturated fatty acids. However, other plasma risk factors linked to cardiovascular disease are less studied. This study aimed to penetrate the effect of a phospholipid-protein complex (PPC) from Antarctic krill on one-carbon metabolism and production of trimethylamine-*N*-oxide (TMAO) in rats. Male Wistar rats were fed isoenergetic control, 6%, or 11% PPC diets for four weeks. Rats fed PPC had reduced total homocysteine plasma level and increased levels of choline, dimethylglycine and cysteine, whereas the plasma level of methionine was unchanged compared to control. PPC feeding increased the plasma level of TMAO, carnitine, its precursors trimethyllysine and γ-butyrobetaine. There was a close correlation between plasma TMAO and carnitine, trimethyllysine, and γ-butyrobetaine, but not between TMAO and choline. The present data suggest that PPC has a homocysteine lowering effect and is associated with altered plasma concentrations of metabolites related to one-carbon metabolism and B-vitamin status in rats. Moreover, the present study reveals a non-obligatory role of gut microbiota in the increased plasma TMAO level as it can be explained by the PPC’s content of TMAO. The increased level of carnitine and carnitine precursors is interpreted to reflect increased carnitine biosynthesis.

## 1. Introduction

Fish oil, enriched by long-chain *n*-3 PUFAs as eicosapentaenoic acid (EPA) and docosahexaenoic acid (DHA), is reported to have protective roles against arteriosclerotic- and cardiovascular diseases [1,2]. It has lately been suggested that some of the beneficial influence of seafood on health could arise from the protein fraction, and studies in rodents have shown that a number of fish hydrolysates have lipid lowering and antioxidant effects [3,4]. Antarctic krill (*Euphausia superba*) has a high protein and lipid content, and has mainly been utilized as a novel source of *n*-3 PUFAs. While krill supplements have been shown to reduce plasma lipid levels in rodent and human studies [5,6], the effect on other risk factors involved in cardiovascular disease has not been analyzed.

**Figure 1:**
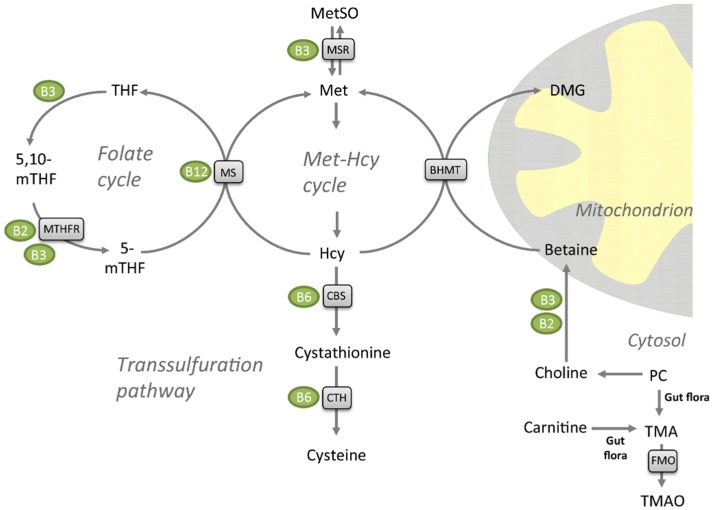
Metabolites of the folate cycle, transsulfuration pathway, and methionine (Met)-homocysteine (Hcy) cycle. Enzymes are shown in grey boxes, and their vitamin co-factors in green circles. Abbreviations: PC, phosphatidylcholine; MetSO, methionine sulfoxide, TMA, trimethylamine; TMAO, trimethylamine-*N*-oxide; FMO, flavin-containing monooxygenase; BHMT, betaine homocysteine methyltransferase; DMG, dimethylglycine; CTH, cystathionine gamma lyase; CBS, cystathionine beta-synthase; MS, methionine synthase/5-methyltetrahydrofolate-homocysteine methyltransferase (MTR); THF, tetrahydrofolate; 5-mTHF, 5-methyltetrahydrofolate; 5,10-mTHF, 5,10-methylenetetrahydrofolate; MTHFR, methylenetetrahydrofolate reductase (NAD(P)H); MSR, methionine synthase reductase.

Elevated plasma total homocysteine (tHcy) increases the likelihood of endothelial cell injury and has been implicated as an independent risk factor for atherosclerotic and cardiovascular disease [7], although its direct association to ischemic stroke has been disputed in recent meta-analyses [8,9]. Plasma tHcy have further been demonstrated to be inversely associated to plasma levels of folate (vitamin B9), cobalamin (vitamin-B12) and pyroxidal phosphate (vitamin B6), the three vitamins involved in the conversion of homocysteine (Hcy) to methionine (Met) or cysteine [10,11]. However, vitamin B interventions have not been able to prevent cardiovascular events [12]. Hcy, a sulfur-containing, non-protein amino acid, is not obtained from the diet—it is generated from *S*-adenosylhomocysteine (SAH) via the *S*-adenosyhomocysteine hydrolase during methylation processes in the body [13]. Remethylation of Hcy back to Met is catalyzed by Met synthase (MS) with 5-methyltetrahydrofolate (5-mTHF) as the methyl donor [14,15]. Cobalamin (vitamin B12) is a cofactor in this enzymatic reaction (Figure 1). This step takes place in all tissues, but in liver and kidney Hcy can also be remethylated to Met by the zinc metalloenzyme betaine-Hcy S-methyltransferase (BHMT) [16,17,18]. Betaine is the methyl donor for BHMT and the products are Met and dimethylglycine (DMG). Met can further be catabolized to Met sulfoxide by the niacin (vitamin B3)-dependent Met sulfoxide reductase A (MSR) [19]. Finally, Hcy catabolism through the transsulfuration pathway to form cysteine is carried out by two pyridoxine (vitamin B6)-dependent enzymes, cystathionine-beta synthase (CBS) and cystathionine gamma-lyase (CTH). Hcy has been suggested to promote atherosclerosis by elevating levels of reactive oxygen species [20], as well as through effects on lipoprotein metabolism [21]. Dietary interventions influencing both Hcy and plasma lipid levels could potentially be beneficial for health. Both *n*-3 polyunsaturated fatty acids (PUFAs) [22], and diets with different amino acid composition [23], have been reported to reduce serum tHcy levels.

The majority of the *n*-3 PUFAs in krill oil is associated with phospholipids, in particular phosphatidylcholine (PC), while in fish oil the PUFAs are in the form of triacylglycerol (TAG) or fatty acid ethyl esters. The phospholipid PC is the major dietary source of choline and it was recently reported that microbiota metabolizes choline to form trimethylamine (TMA), which can be oxidized by the vitamin-B2 dependent enzyme flavin adenine monomaine oxidase (FMO) in the liver to produce trimethylamine-*N*-oxide (TMAO) (Figure 1) [24,25]. Metabolism of dietary carnitine by intestinal microbiota has also been found to produce TMAO [26]. Intestinal microbial metabolism of PC and carnitine has been found to promote arteriosclerosis and is associated with an increased risk of incident major adverse cardiovascular events, but only among subjects with high TMAO levels [25,27]. Nevertheless, it is important to note that TMAO has beneficial effects in marine animals; TMAO is a common and compatible osmolyte in muscle tissues that protects proteins against various destabilizing forces [28]. It is reported that TMAO counteract the toxic effects of urea and ammonia on proteins [29], and may also depress the freezing point and counteract the effect of hydrostatic pressure of enzyme function in deep-sea animals [28,30,31].

The aim of the present study was to provide insight into effects of a phospholipid-protein complex (PPC) from krill with emphasis on diet-related changes in plasma tHcy and TMAO. Thus, we measured the effect of PPC on the Met-Hcy cycle, transsulfuration pathway, vitamin B status and hepatic gene expression, as well as plasma levels of choline, betaine, carnitine, and carnitine precursors, all linked to TMAO generation.

## 2. Results

### 2.1. Metabolites of the Methionine-Homocysteine Cycle

**Figure 2:**
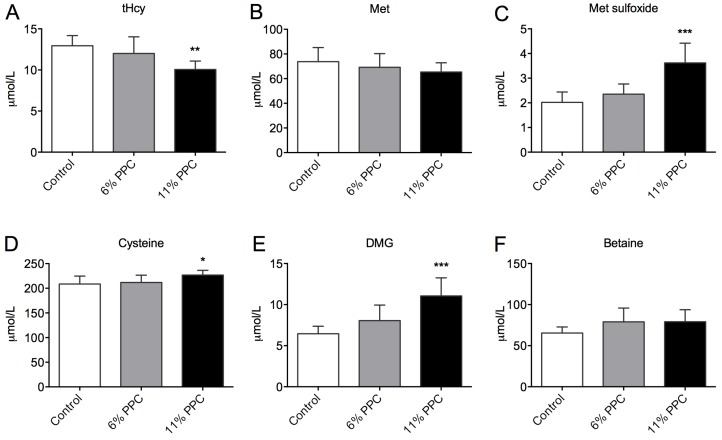
Plasma levels of metabolites of the transsulfuration and homocysteine remethylation pathways. Rats were fed either a control diet (2% soy oil, 8% lard, 20% casein), or an experimental diet where casein and lard were replaced with phospholipid-protein complex (PPC) at 6% or 11% (wt%) for 4 weeks. Total homocysteine (tHcy; **A**), methionine (Met; **B**), Met sulfoxide (**C**), cysteine (**D**), dimethylglycine (DMG; **E**), and betaine (**F**) were measured in fasting plasma samples. Values shown are means with standard deviation (*n* = 6). One-way analysis of variance (ANOVA) with Dunnet’s *post hoc* test was used to determine values significantly different from control (* *p* < 0.05, ** *p* < 0.01, *** *p* < 0.001).

The plasma levels of tHcy (Figure 2A) were significantly reduced by 11% PPC feeding compared to control. The plasma levels of Met did not differ between the groups (Figure 2B), whereas the plasma levels of Met sulfoxide were significantly increased in the 11% PPC-fed rats (Figure 2C). Along the transsulfuration pathway the plasma level of cysteine demonstrated a small, but significantly increase in the 11% PPC-fed rats compared to control (Figure 2D). However, there was no inverse correlation between the tHcy and cysteine plasma levels (*R* = −0.051, *p* = 0.851, *n* = 16). Along the choline oxidation pathway, the PPC-fed rats had higher levels of plasma DMG (Figure 2E), while the plasma level of betaine did not differ statistically between the groups (Figure 2F). A tendency to negative correlation was observed between plasma levels of DMG and tHcy (*R* = −0.454, *p* = 0.077, *n* = 16). No significant changes in the hepatic gene expression of enzymes involved in Hcy remethylation and sulfoxation or transsulfuration, or choline biosynthesis were observed (Table S1).

### 2.2. Vitamin B Levels

**Figure 3:**
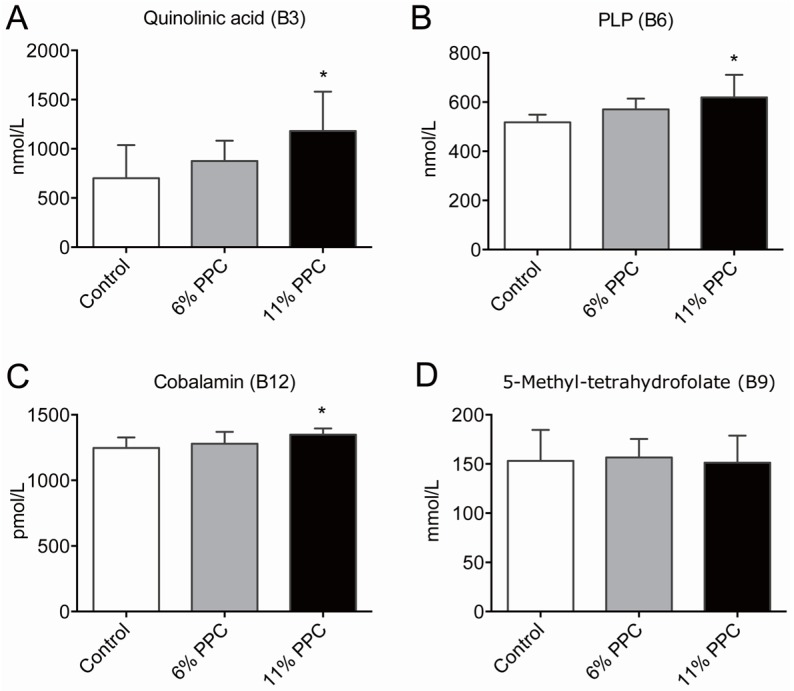
Plasma levels of B-vitamins and derivatives. Male Wistar rats were fed either a control diet (2% soy oil, 8% lard, 20% casein), or an experimental diet where casein and lard were replaced with phospholipid-protein complex (PPC) at 6% or 11% (wt. %) for 4 weeks. Quinolinic acid (vitamin B3, **A**), pyridoxal phosphate (PLP; vitamin B6, **B**), cobalamin (vitamin B12, **C**), and 5-methyltetrahydrofolate (5-mTHF; **D**) were measured in fasting plasma samples. Values shown are means with standard deviation (*n* = 6). One-way analysis of variance (ANOVA) with Dunnet’s *post hoc* test was used to determine values significantly different from control (* *p* < 0.05).

Among the B-vitamins, higher levels of plasma quinolinic acid (vitamin B3, Figure 3A), pyroxidal phosphate (PLP; vitamin B6, Figure 3B), and cobalamin (vitamin B12, Figure 3C), was measured in the 11% PPC-fed rats compared to controls. The plasma levels of 5-mTHF (activated form of folate/vitamin B9) did not differ between the groups (Figure 3D). In addition, no differences in plasma riboflavin (vitamin B2), flavin mononucleotide (FMN; a biomolecule produced from riboflavin), nicotinamid (vitamin B3), *N*’-methylnicotinamid (metabolite of nicotinamid), pyridoxic acid (form of vitamin B6), and pyridoxal (form of vitamin B6) were found in the PPC-fed rats *versus* controls (Figure S1).

### 2.3. Choline, Carnitine, Carnitine Precursors and TMAO

**Figure 4:**
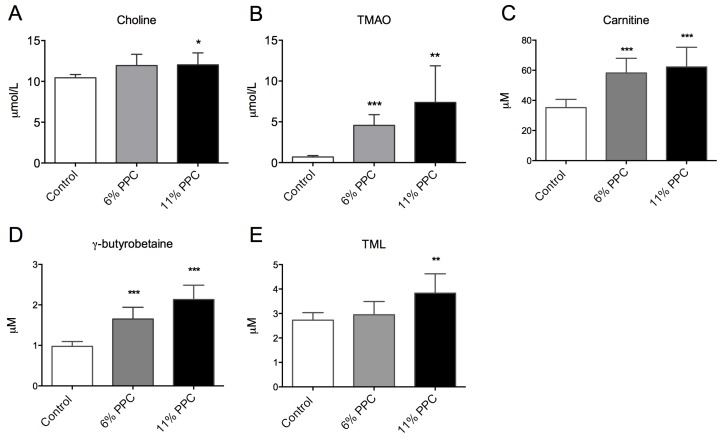
Plasma levels of phosphatidylcholine (PC) degradation products and carnitine precursors. Male Wistar rats were fed either a control diet (2% soy oil, 8% lard, 20% casein), or an experimental diet where casein and lard were replaced with phospholipid-protein complex (PPC) at 6% or 11% (wt%) for four weeks. Choline (**A**), trimethylamine-*N*-oxide (TMAO; **B**), carnitine (**C**), γ-butyrobetaine (**D**) and trimethyllysine (TML; **E**) were measured in fasting plasma samples. Values shown are means with standard deviation (*n* = 6). One-way analysis of variance (ANOVA) with Dunnet’s *post hoc* test was used to determine values significantly different from control (** *p* < 0.01, *** *p* < 0.001).

**Figure 5:**
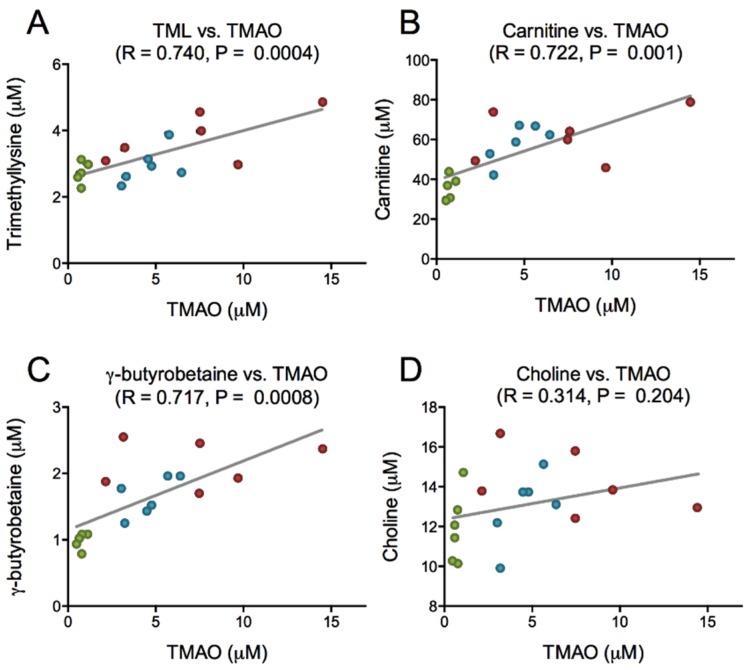
Correlation between trimethylamine-*N*-oxide (TMAO) and intermediates in TMAO synthesis. Plasma levels of TMAO and trimethyllysine (**A**), TMAO and carnitine (**B**), TMAO and γ-butyrobetaine (**C**), and TMAO and choline (**D**), were analyzed by linear regression. Data were obtained as described under legend to Figure 4. Control group is depicted in green, 6% phospholipid-protein complex (PPC) in blue, and 11% PPC in red.

PPC is rich in PC, and is a natural source of TMAO, but has low concentrations of carnitine, carnitine precursors as well as free choline (Table 1). After PPC-feeding the plasma level of choline was increased in the 11% PPC-fed rats compared to the control-fed rats (Figure 4A), whereas the plasma levels of TMAO were increased by both doses of PPC (Figure 4B). The plasma levels of carnitine and the intermediates in carnitine biosynthesis, trimethyllysine (TML) and γ-butyrobetaine, were significantly increased by PPC-feeding compared to control (Figure 4C–E). Further, a close correlation between plasma TMAO and TML, carnitine and γ-butyrobetaine, but not choline was found (Figure 5).

**Table 1:** Contents of free choline, carnitine, carnitine precursors and trimethylamine-*N*-oxide (TMAO) (µmol/100 g) in the control and 11% phospholipid-protein complex (PPC) diets.

Organic Compound	Control ^1^	11% PPC
TMAO	<0.001	12.6
Free choline	17.1	20.9
Betaine	<0.001	3.9
Carnitine	0.25	0.27
γ-Butyrobetaine	<0.001	0.009
TML	<0.001	0.003
TMA ^1^	-	7.06

^1^ Calculated from commercial analysis of phospholipid-protein complex (PPC), and assumed not to be present in the control diet. Abbreviations: PC, phosphatidyl choline; TMA, trimethylamine; TMAO, trimethylamine-*N*-oxide; TML, trimethyllysine.

## 3. Discussion

The role of *n*-3 PUFA supplementation in the regulation of risk factors for cardiovascular disease has been analyzed in numerous studies, and krill oil in particular has many promising effects based on its high bioavailability (reviewed by [32]). We have previously shown that PPC from krill lowers plasma TAG and cholesterol levels in male Wistar rats [33]. The present study focused on plasma parameters where the effects of krill supplements are unknown; tHcy and the newly established risk factor TMAO. We demonstrate that 11% PPC influenced Met-Hcy cycle metabolites including choline and DMG, as well as the vitamin B status, compared to a control diet. In addition, PPC-feeding altered the plasma level of TMAO compared to control. Further, the increase in TMAO positively correlated with plasma carnitine and carnitine precursors.

As hyperhomocysteinemia is an established risk factor for coronary artery disease [7,34,35,36], it was of interest to note that the plasma tHcy level was significantly decreased by PPC supplementation. A recent meta-analysis of intervention studies show that *n*-3 PUFA supplementation is associated with reduced plasma tHcy levels [22], which is in line with our findings. Compared to the control diet, the PPC diets contained high levels of betaine (Table 1), as well as choline from the PC component. A high dietary intake of choline and betaine has been linked to reduction in tHcy levels [37,38]. While a number of studies indicate a lower risk of CVD after long term choline and betaine intake, by the ability to lower inflammatory biomarkers and counteract oxidative stress caused by high Hcy (reviewed in [39]), an association to survival has not been confirmed in cohort studies [40]. In the present study the plasma level of betaine was unchanged while the DMG level increased in the PPC-fed rats compared to controls. This indicates higher activity of BHMT-dependent Hcy remethylation with betaine as methyldonor (Figure 1), supported by a strong tendency to negative correlation between plasma levels of tHcy and DMG. As DMG is implicated in the regulation of glucose metabolism, and low DMG levels are associated with higher blood glucose levels [41], dietary regulation of this factor could prove useful in the prevention of diabetes mellitus type 2.

Vitamin cofactors are important in the different pathways regulating tHcy. The plasma content of quinolinic acid, a metabolite associated with vitamin B3, was higher in the 11% PPC group, and could increase enzyme activities of BHMT and MSR. The lower level of tHcy could also be the consequence of increased activity of MS as a higher plasma level of cobalamin (vitamin B12) resulting after PPC-feeding. Thus, Hcy remethylation involving both 5-mTHF and betaine as methyl donors was potentially increased by 11% PPC.

Likewise, the higher level of pyridoxal phosphate, an active metabolite of vitamin-B6, could increase enzyme activities of CBS and CTH, activating the transsulfuration pathway important in the Hcy elimination process. In the present study, the product of this reaction, plasma cysteine, was significantly higher in the 11% PPC-fed rats than in controls. In rats supplemented with DHA from tuna oil, plasma tHcy was reduced partly through upregulation of hepatic transsulfuration [42]. Thus, the *n*-3 PUFA component in the PPC diet could have contributed to the reduction of tHcy through effects on transsulfuration. However, as there was no negative correlation between plasma levels of tHcy and cysteine, the transsulfuration pathway could be less important than remethylation in the regulation of plasma tHcy after PPC-feeding.

An elevated plasma level of microbiota-derived TMAO has been found to promote arteriosclerosis in animal studies, and is associated with increased risk of adverse cardiovascular events. However, as seafood contains high levels of TMAO [43], and is generally considered beneficial for health, this is somewhat contradictory. Crustaceans are a natural source of TMAO, as TMAO is synthesized directly from choline *in vivo* and functions to counteract protein-destabilization in deep-sea organisms [28,31,44]. Although the necessary enzymes for this process have been identified in mammals [45], TMAO is formed in the liver from gut-derived trimethylamine (TMA) generated from dietary choline and carnitine [25,26]. In the present study, PPC-feeding increased the plasma levels of TMAO, choline, carnitine and its precursors TML and γ-butyrobutaine (Figure 4). As the PPC contain low amounts of γ-butyrobetaine, TML, and carnitine, but high amounts of TMAO and TMA, the increased plasma level of TMAO in the PPC-fed rats could be due to direct dietary uptake (Table 1). In addition, PPC’s content of PC (17 g PC/100 g 11% PPC diet) may have contributed to plasma TMAO levels through microbial activity. However, there was no correlation between plasma choline and TMAO in rats fed PPC (Figure 5). Moreover, unpublished material from a recently conducted intervention study with phospholipids, predominantly PC, from herring roe (1.7 g PL/day for two weeks) showed no increase in plasma TMAO levels in healthy young adults (Table S2) [46]. Importantly, there seem to be a link between TMAO, choline and betaine in their prognostic value [47], and positive associations to cardiovascular risk are more prominent in diabetic subjects, indicating that control of osmolyte retention in tissues could be important [48]. Further studies are needed to determine the effect of seafood-derived TMAO on these processes.

As carnitine is a precursor of TMAO [49], the close positive correlation between plasma TMAO and carnitine and its precursors was intriguing. However, in the current study, it was not possible to connect this to dietary intake of carnitine. The observation could suggest a concomitant increase in plasma TMAO, due to dietary intake, and carnitine, due to increased biosynthesis. Krill oil has previously been shown to increase the plasma carnitine level, but not the level of carnitine precursors in mice [50]. Interestingly, while choline-supplementation was shown to reduce serum carnitine levels in humans [51], betaine increases carnitine production in mice [52]. This indicates that the higher betaine-level in the PPC diets could result in stimulated carnitine biosynthesis. Thus, the increased plasma levels of both carnitine and carnitine precursors in the PPC-fed rats could partly be due to PPC’s content of *n*-3 PUFA-rich oil, and partly an increased betaine intake. l-Carnitine supplementation improves lipid metabolism in obese rats [53], but does not affect cardiovascular risk in humans [54]. Further studies are needed to determine a possible beneficial effect of increased carnitine biosynthesis on metabolic disease.

## 4. Materials and Methods

### 4.1. Animals and Diets

The animal study was conducted according to the Guidelines for the Care and Use of Experimental Animals, and in accordance with the Norwegian legislation and regulations governing experiments using live animals. The Norwegian State Board of Biological Experiments with Living Animals approved the protocol (Permit number 2013-5324). All efforts were made to optimize the animal environment, and minimize suffering.

Male Wistar rats, aged 5 to 6 weeks (Taconic Tornbjergvej facility, Elby, Denmark), were randomized and housed pair wise in open cages (*n* = 6 rats per group). They were kept under standard laboratory conditions with temperature 22 ± 1 °C, dark/light cycles of 12/12 h, relative humidity 43% ± 5%, and 20 air changes per hour. The rats were acclimatized under these conditions for one week prior to study start, with free access to standard chow and water.

The rats were fed *ad libitum* for 4 weeks on a 10% fat diet, either as a control diet (2% soy oil, 8% lard, 20% casein, wt.%) or an experimental diet, where casein and lard were replaced with PPC at 6% or 11% (wt.%). See Ramsvik *et al.* for a detailed description of the diet ingredients [33]. Krill PPC, an Antarctic krill meal from Euphausia superba (RIMFROST GENUINE^®^), was delivered by Olympic Seafood AS (Fosnavaag, Norway). The PPC consisted of 46.4% protein and 45.7% fat, and contained 39.0 g phosphatidylcholine, 13 g eicosapentaenoic acid (EPA, C20:5*n*-3), and 7.9 g docosahexaenoic acid (DHA, C22:6*n*-3) per 100 g extracted fat. The fatty acid composition of the PPC and the amino acid composition in casein and the PPC are presented in Table 2 and Table 3, respectively. Feed intake and weight gain were determined twice a week. All rats were killed on day 28. They were anesthetized by inhalation of 2% Isofluorane (Forane, from Abbot Laboratories Ltd., Abbott Park, IL, USA) and thoracotomy, cardiac puncture, and exsanguination was performed. Plasma and liver samples were stored at −80 °C.

**Table 2:** Lipids and fatty acid composition (wt%) of the phospholipid-protein complex (PPC).

Fatty Acid	PPC ^1^
C14:0	6.7
C16:0	16.0
C18:0	0.9
C20:0	<01
C22:0	<0.1
C16:1*n-*7	2.6
C18:1(*n*-9) + (*n*-7) + (*n*-5)	12.7
C20:1(*n-*9) + (*n*-7)	0.6
C22:1(*n-*11) + (*n-*9) + (*n*-7)	0.3
C24:1*n-*9	<0.01
C18:2*n-*6 (LA)	1.6
C18:3*n-*6	0.1
C20:2*n-*6	<0.1
C20:3*n-*6	<0.1
C20:4*n-*6 (AA)	0.1
C22:4*n-*6	<0.1
C18:3*n-*3 (ALA)	3.2
C18:4*n-*3	6.9
C20:3*n-*3	0.1
C20:4*n-*3	0.5
C20:5*n-*3 (EPA)	13.0
C21:5*n-*3	0.5
C22:5*n-*3 (DPA)	0.4
C22:6*n-*3 (DHA)	7.9
∑SFAs	23.6
∑MUFAs	16.2
∑PUFAs	35.1
*n-*6 PUFAs	1.8
*n-*3 PUFAs	32.5
*Sum of fatty acids*	74.9
**Lipids**	
Total polar lipids	46.9
Total neutral lipids	53.1
*Sum of lipids*	100

^1^ Fat (wt%) Bligh & Dyer, g/100 g extracted lipids. Abbreviations: AA, arachidonic acid; ALA, alpha linolenic acid; DHA, docosahexaenoic acid; DPA, docosapentaenoic acid; EPA, eicosapentaenoic acid; LA, linoleic acid; MUFAs, monounsaturated fatty acids; PUFAs, polyunsaturated fatty acids; SFAs, saturated fatty acids.

**Table 3:** Amino acid composition in casein and the phospholipid-protein complex (PPC).

Amino acid ^1^	Dietary Component	
	Casein ^2^	PPC ^3^
Aspartic acid	6.5	10.6
Glutaminic acid	20.8	12.7
Hydroksyproline	-	<0.01
Serine	5.4	4.8
Glycine	1.8	4.6
Histidine	2.6	2.7
Arginine	3.6	6.2
Threonine	3.8	5.4
Alanine	2.6	5.1
Proline	11.7	4.5
Tyrosine	5.3	4.7
Valine	5.7	5.8
Methionine	2.6	3.3
Isoleucine	4.8	6.5
Leucine	8.8	8.8
Phenylalanine	5.0	5.4
Lysine	7.4	9.0
Cysteine	0.4	-
Tryptophan	1.2	-
Methionine/glycine	1.4	0.7
Lysine/arginine	2.1	1.5

^1^ g amino acid/100 g of protein; ^2^ Values given by the manufacturer; ^3^ Analyzed values.

### 4.2. Biochemical Analyses

Plasma trimethyllysine (TML), choline, betaine, DMG and TMAO were analyzed by liquid chromatography-mass spectrometry [27,46]. Met, Met sulfoxide, tHcy, cystathione, and cysteine were analyzed by gas chromatography-mass spectrometry [55]. Vitamin B status (flavin mononucleotide (FMN), riboflavin, nicotinic acid, quinolinic acid, pyridoxal phosphate (PLP), pyridoxal, pyridoxic acid, folate, and cobalamin) was measured as previously described [55].

### 4.3. Statistical Analysis

Data sets were analyzed using Prism Software (Graph-Pad Software, San Diego, CA, USA) to determine statistical significance. The results are shown as means of 6 animals per group with their standard deviations. Normal distribution was determined by the Kolmogorov-Smirnov test (with Dallal-Wilkinson-Lilliefor corrected *p*-value). One-way analysis of variance (ANOVA) with Dunnet’s *post hoc* test was used to evaluate statistical differences between groups. Unpaired *t*-test test was used for the gene analysis. Pearson’s correlation coefficient (*R*) was calculated to measure linear dependence between plasma metabolites. *p*-Values < 0.05 were considered significant.

## 5. Conclusions

A phospholipid-protein complex from krill reduced plasma tHcy levels, possibly associated with the *n*-3 PUFA, choline and betaine content of the diet, which suggest a positive health effect of PPC. On the other hand, a higher plasma level of TMAO, as observed in the PPC-fed rats, can potentially increase the risk of cardiovascular disease. Importantly, this increase seemed to be mainly due to direct uptake of TMAO from the PPC and not through microbiota-dependent mechanisms. As seafood has been shown to improve cardiovascular health, beneficial effects could counteract the possible negative effect of TMAO uptake. Whether TMAO from seafood in fact has negative effects when not generated from TMA produced in the intestines has yet to be determined. Although there was a significant correlation between plasma carnitine, TML and TMAO in rats, this was not due to TMA generation from carnitine and TML by microbiota, but rather a stimulation of carnitine biosynthesis dependent on PPC-intake.

## Supplementary Materials

**Figure S1**: Plasma levels of riboflavin, FMN, nicotinamid, *N*’-methylnicotinamid, pyridoxic acid, pyridoxal, and folate.

**Table S1**: Hepatic expression of genes involved in choline metabolism, the methionine-homocysteine cycle, and homocysteine transsulfuration.

**Table S2**: Trimethylamine-*N*-oxide (TMAO) levels in plasma after 2 weeks supplementation with phosphatidylcholine from herring roe in healthy young adults (unpublished results).
