# Supplementary material for: A Phospholipid-Protein Complex from Antarctic Krill Reduced Plasma Homocysteine Levels and Increased Plasma Trimethylamine-N-Oxide (TMAO) and Carnitine Levels in Male Wistar Rats
Source: Mar Drugs. 2015 Sep 8;13(9):5706–21. doi: 10.3390/md13095706 (PMC4584349; doi:10.3390/md13095706)
Supplement: Supplementary file 1 [file marinedrugs-13-05706-s001.docx]

Supplementary Information

Gene Expression Analysis

Total cellular RNA was purified from frozen liver samples, and cDNA was produced as previously described [1]. Real-time PCR was performed with Sarstedt 384 well multiply-PCR Plates (Sarstedt Inc., Newton, NC, USA) on the following genes, using probes and primers from Applied Biosystems (Waltham, MA, USA): phosphatidylethanolamine *N*-methyltransferase (*Pemt*, Rn00564517_m1), betaine-homocysteine methyltransferase (*Bhmt*, Rn00578255_m1), methionine synthase/5-methyltetrahydrofolate-homocysteine methyltransferase (*Ms*, Rn00578368_m1), methionine sulfoxide reductase A (*Msra*, Rn00584008), methionine sulfoxide reductase B2 (*Msrb2*, Rn01765104_m1), cystathionine beta-synthase (*Cbs*, Rn00560948_m1), cystathionase/cystathionine gamma-lyase (*Cth*, Rn00567128_m1), 5,10-methylenetetrahydrofolate reductase (*Mthfr*, Rn01515583_m1).

Three different reference genes were included: 18*s* (Kit-FAM-TAMRA (Reference RT-CKFT-18s)) from Eurogentec (Liège, Belgium), glyceraldehyde-3-phosphate dehydrogenase (*Gapdh*, Mm99999915_g1) from Applied Biosystems, and ribosomal protein, large, P0 (*Rplp0*, Gene ID 11837) from Thermo Fisher Scientific. All gene-runs included standard curves using either cDNA generated from universal rat reference RNA (URRR, Agilent Technologies, Santa Clara, CA, USA) or pooled cDNA as template. The NormFinder software was used to evaluate the stability of the reference genes [2], and based on this; the concentration of each analysed gene was normalized to the concentration of *Rplp0* in each sample. Finally, expression values relative to the control group were calculated.

**Table S1.** Hepatic gene expression in Wistar rats on a control or a 11% phospholipid-protein complex (PPC)-diet.

|  | **Diet** | |  |
| --- | --- | --- | --- |
| **Gene symbol** | **Control ^1^** | **11% PPC ^1^** | ***p*-value ^2^** |
| *Pemt* | 1.0 ± 0.20 | 0.92 ± 0.38 | 0.681 |
| *Ms* | 1.0 ± 0.20 | 0.94 ± 0.42 | 0.761 |
| *Mthfr* | 1.0 ± 0.40 | 0.61 ± 0.39 | 0.142 |
| *Msra* | 1.0 ± 0.15 | 0.97 ± 0.35 | 0.865 |
| *Msrb2* | 1.0 ± 0.16 | 0.86 ± 0.30 | 0.366 |
| *Cth* | 1.0 ± 0.37 | 0.95 ± 0.41 | 0.829 |
| *Cbs* | 1.0 ± 0.26 | 1.09 ± 0.38 | 0.679 |
| *Bhmt* | 1.0 ± 0.38 | 1.21 ± 0.50 | 0.468 |

^1^ Mean values relative to control ± standard deviation (SD) are shown (*n* = 5–6). ^2^ Statistically significant difference was calculated using unpaired *t*-test (*p* < 0.05). Abbreviations: *Pemt*, phosphatidylethanolamine
*N*-methyltransferase; *Ms*, 5-methyltetrahydrofolate-homocysteine methyltransferase; *Mthfr*,
5,10-methylenetetrahydrofolate reductase; *Msra*, methionine sulfoxide reductase A; *Msrb2*, methionine sulfoxide reductase B2; *Cth*, cystathionase/cystathionine gamma-lyase; *Cbs*, cystathionine beta-synthase; *Bhmt*, betaine-homocysteine methyltransferase.

**Table S2.** Trimethylamine *N*-oxide (TMAO) levels (μM) in plasma after 2 weeks supplementation with phosphatidylcholine from herring roe in healthy young adults ^1^.

| **Participant number** | **Baseline** | **End of study** |
| --- | --- | --- |
| 1 | 1.8 | 2.3 |
| 2 | 3.4 | 1.8 |
| 3 | 1.9 | 6.4 |
| 4 | 2.2 | 13.4 |
| 5 | 1.3 | 2.3 |
| 6 | 5.0 | 2.0 |
| 7 | 29.3 | 35.3 |
| 8 | 3.2 | 2.7 |
| 9 | 3.4 | 3.5 |
| 10 | 2.4 | 1.7 |
| 11 | 3.8 | 2.9 |
| 12 | 2.8 | 2.8 |
| 13 | 1.5 | 8.0 |
| 14 | 7.4 | 2.3 |
| 15 | 2.8 | 2.5 |
| 16 | 3.0 | 2.4 |
| 17 | 3.7 | 4.1 |
| 18 | 23.9 | 6.7 |
| 19 | 7.4 | 19.1 |
| Average | 5.7 | 6.1 |
| Standard deviation | 7.2 | 8.0 |
| *p*-value (pairwise *t*-test) |  | 0.736 |

^1^ Daily herring roe dose consisted of 1.7 g phospholipids, of which 75% were phosphatidylcholine [3].


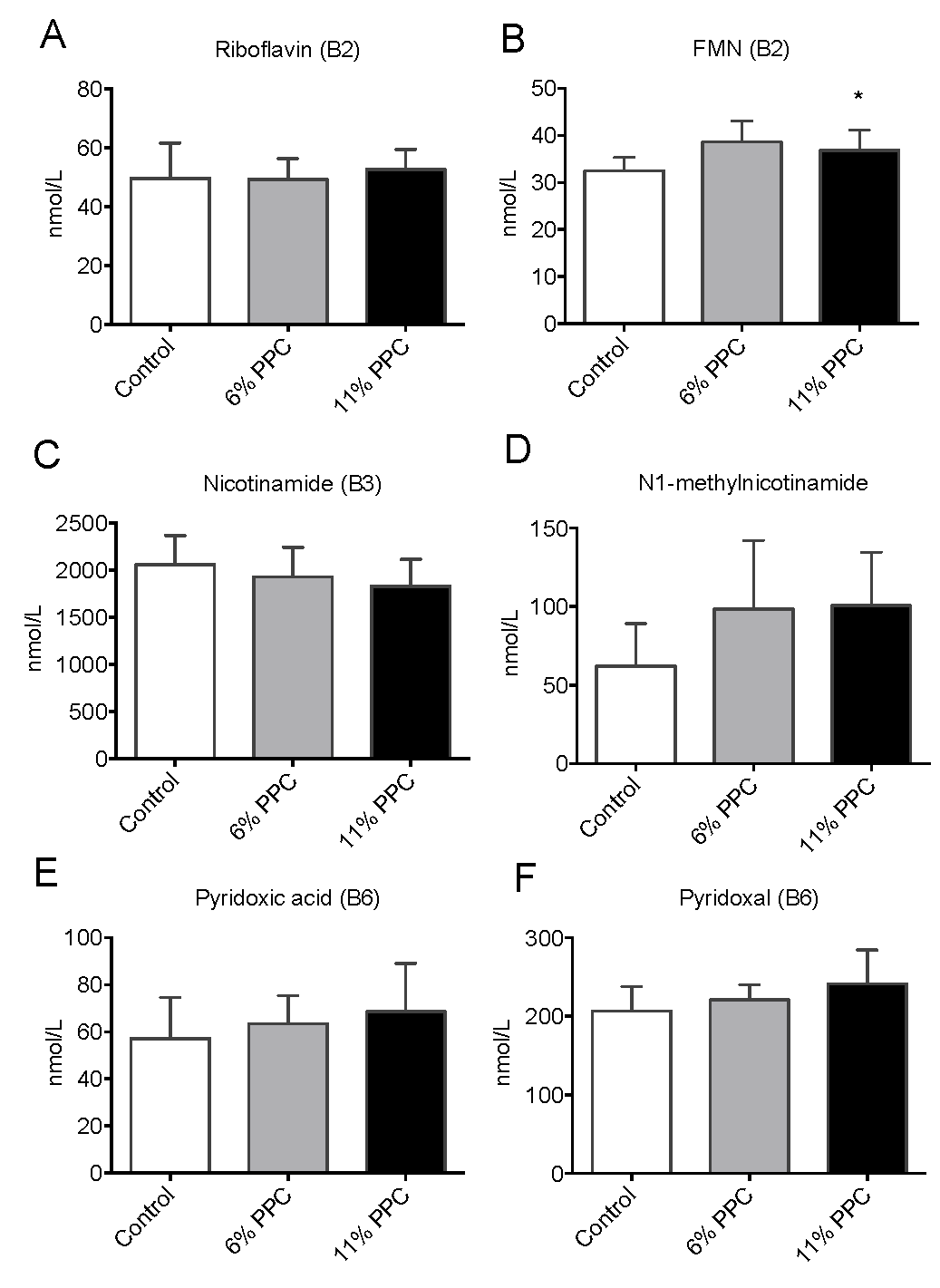


**Figure S1.** *Cont.*


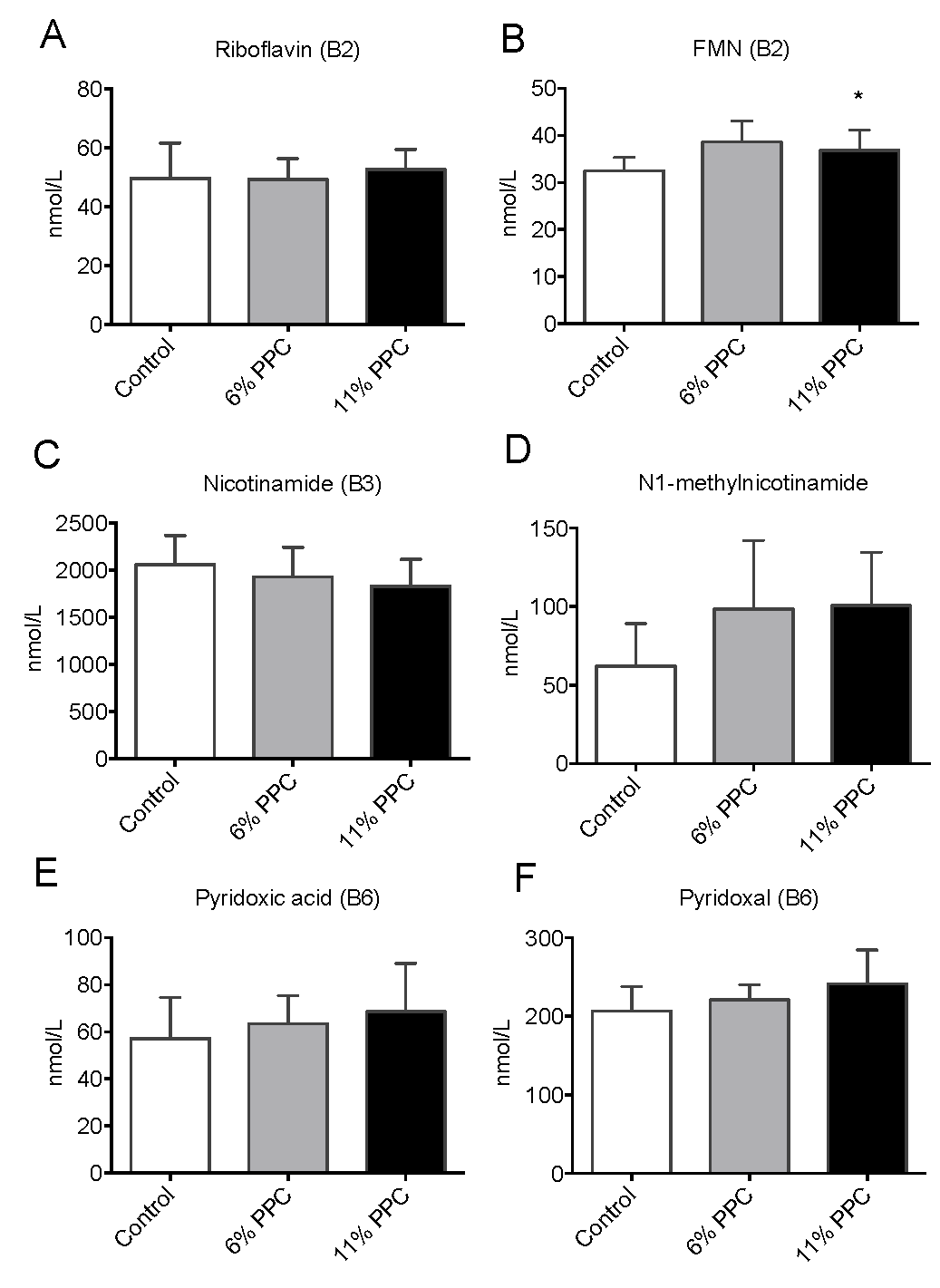


**Figure S1.** Plasma levels of B-vitamins and derivatives. Male Wistar rats were fed either a control diet (2% soy oil, 8% lard, 20% casein), or an experimental diet where casein and lard were replaced with phospholipid-protein complex (PPC) at 6% or 11% (wt%) for 4 weeks. Riboflavin (vitamin B2, **A**), flavin mononucleotide (FMN; vitamin B2, **B**), nicotinamide (vitamin B3, **C**), *N*1-methylnicotinamide (vitamin B3, **D**), pyridoxic acid (vitamin B6, **E**), and pyridoxal (vitamin B6, **F**) were measured in fasting plasma samples. Values shown are means with standard deviation (n = 6). One-way analysis of variance (ANOVA) with Dunnet’s *post hoc* test was used to determine values significantly different from control (***** *p* < 0.05).

**References**

1. Vigerust, N.F.; Cacabelos, D.; Burri, L.; Berge, K.; Wergedahl, H.; Christensen, B.; Portero-Otin, M.; Viste, A.; Pamplona, R.; Berge, R.K.; *et al.* Fish oil and 3-thia fatty acid have additive effects on lipid metabolism but antagonistic effects on oxidative damage when fed to rats for 50 weeks.
*J. Nutr. Biochem.* **2011**, *23*, 1384–1393.

2. Andersen, C.L.; Jensen, J.L.; Orntoft, T.F. Normalization of real-time quantitative reverse transcription-PCR data: A model-based variance estimation approach to identify genes suited for normalization, applied to bladder and colon cancer data sets. *Cancer. Res.* **2004**, *64*, 5245–5250.

3. Chiuve, S.E.; Giovannucci, E.L.; Hankinson, S.E.; Zeisel, S.H.; Dougherty, L.W.; Willett, W.C.; Rimm, E.B. The association between betaine and choline intakes and the plasma concentrations of homocysteine in women. *Am. J. Clin. Nutr.* **2007**, *86*, 1073–1081.

© 2015 by the authors; licensee MDPI, Basel, Switzerland. This article is an open access article distributed under the terms and conditions of the Creative Commons Attribution license (http://creativecommons.org/licenses/by/4.0/).
